# Monitoring Glutathione Content of the Endoplasmic Reticulum under Scrap Leather-Induced Endoplasmic Reticulum Stress via an Endoplasmic Reticulum-Targeted Two-Photon Fluorescent Probe

**DOI:** 10.1021/acs.analchem.4c04157

**Published:** 2024-10-30

**Authors:** Xinjian Song, Xumei Wang, Yan Wang, Yiqian Hao, Chenchen Li, Li Chai, Haixian Ren, Jianbin Chen, Wei Hu, Tony D. James

**Affiliations:** a Hubei Key Laboratory of Biological Resources Protection and Utilization, School of Chemical and Environmental Engineering, Hubei Minzu University, Enshi, Hubei 445000, China; b Department of Chemistry, Xinzhou Normal University, Xinzhou, Shanxi 034000, China; c Department of Chemistry, University of Bath, Bath BA27AY, United Kingdom; d School of Chemistry and Chemical Engineering, Henan Normal University, Xinxiang 453007, China; e School of Chemistry and Chemical Engineering, Qilu University of Technology (Shandong Academy of Sciences), Jinan, Shandong 250353, China

## Abstract

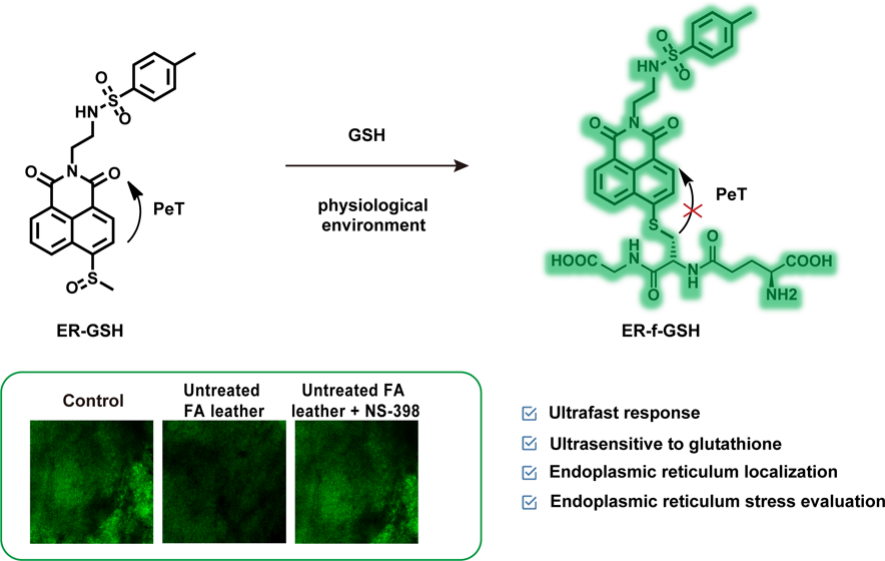

Maintaining tissue homeostasis necessitates the coordinated efforts of various cell types to regulate inflammation. Endoplasmic reticulum (ER) stress, a hallmark of inflammation, exacerbates tissue pathology in various human diseases. Glutathione (GSH), a pivotal regulator of cellular redox balance, controls disulfide bond formation in the ER, thereby shielding cells from oxidative stress. In this study, we developed a two-photon fluorescent probe, ER-GSH, with specific ER targeting and demonstrated its high sensitivity and rapid response to GSH. Experiments conducted on BV2 cells and a mice model of neuroinflammation induced by scrap leather revealed that inflammatory reactions led to ER stress and a substantial reduction in GSH levels. Notably, the anti-inflammatory drug NS-398 effectively inhibited cell inflammation and ER stress by maintaining GSH levels. These findings underscore the potential therapeutic significance of modulating GSH levels to alleviate the impact of neuroinflammation.

## Introduction

The endoplasmic reticulum (ER) is a pivotal organelle in eukaryotic cells, responsible for vital biological processes such as protein synthesis, folding, and modification. Within the ER, newly synthesized proteins undergo folding processes to attain functional conformations.^1,2^ However, the functionality of the ER can be compromised when cells encounter external pressures or internal perturbations, such as oxidative stress, nutrient deprivation, or drug influences, leading to dysregulation of protein folding and aberrant accumulation.^3^ The occurrence of ER stress triggers a cascade of intricate signaling pathways within the cell, including IRE1, PERK, and ATF6 pathways. Activation of these pathways instigates the unfolded protein response (UPR) aimed at restoring ER function and shielding cells from stress-induced damage.^4^ Nevertheless, when stress becomes excessively severe or prolonged, the UPR may fail to effectively alleviate the stress, culminating in cellular apoptosis or disease development. Hence, accurate and timely detection of ER stress is paramount for a comprehensive understanding of cellular responses to environmental changes.

Cells contain glutathione (GSH), consisting of glutamic acid, cysteine, and glycine, as the most abundant thiol-containing molecule.^5,6^ In the ER cavity’s partially oxidized environment, GSH predominantly exists in the oxidized form (ER GSH reduction potential is −150 mV, significantly greater than the −260 mV in the cytoplasm).^7,8^ Nevertheless, its level controls disulfide bond formation in the ER and plays a crucial role in maintaining the cellular redox state and shielding against oxidative stress.^9^ Hence, this study focuses on accurately assessing changes in GSH levels within the cellular ER. Fluorescence probes offer various benefits, such as high sensitivity, selectivity, simplicity, low cost, and accuracy.^10−12^ They facilitate the detection of changes in fluorescence intensity or maximum emission wavelength before and after a reaction, enabling specific analyte recognition.^13^ Utilizing two-photon confocal imaging technology with fluorescent probes allows the real-time, *in situ* detection of specific target substances in a biological system.^14,15^ This technology facilitates high-resolution, depth-enabled monitoring of physiological and pathological processes *in vivo*, providing an effective approach to observe GSH level fluctuations during ER stress.^16^

With this research we devised and synthesized a two-photon GSH probe named ER-GSH, incorporating an ER-targeting group. ER-GSH also incorporates a naphthalimide derivative as a two-photon fluorophore, methyl sulfoxide as the GSH recognition group, and *p*-toluenesulfonic acid as the ER-targeting group.^17^ The observed results confirmed the outstanding GSH responsive nature of ER-GSH, with two-photon active cross sections (δΦ) of 37 and 180 GM (Goeppert-Mayer, unit of two-photon active cross section) before and after reaction, respectively (λ_ex_ = 830 nm). Additionally, the one-photon fluorescence intensity increased by 304-fold within 40 min. Hence, with this research, we could use ER-GSH to investigate an ER stress model induced by scrap leather. The findings revealed that ER stress resulted in a reduction in cellular GSH concentrations, while the fluorescence intensity significantly increased following the introduction of the nonsteroidal anti-inflammatory agent NS-398. These outcomes highlight that scrap leather induces ER stress in cells due to inflammation, causing a disturbance in the ER’s redox balance.

## Results and Discussion

### Rational Design of Probe ER-GSH

Herein, we synthesized ER-GSH, a GSH fluorescent probe, with naphthalimide as the two-photon fluorophore and methyl sulfoxide as the trigger group. The sulfoxide group present in ER-GSH can quench the fluorescence of the fluorophore via photoinduced electron transfer (PeT).^18^ The mechanism for the interaction of ER-GSH with GSH was confirmed using high-resolution mass spectrometry (HRMS). The peak at *m*/*z* = 700.1735 was ascribed to the characteristic peak of the reaction product ER-f-GSH [M + H]^+^, and the peak at *m*/*z* = 457.0833 associated with the reactant ER-GSH [M + H]^+^ disappeared (Figure S5).

**Figure 1:**
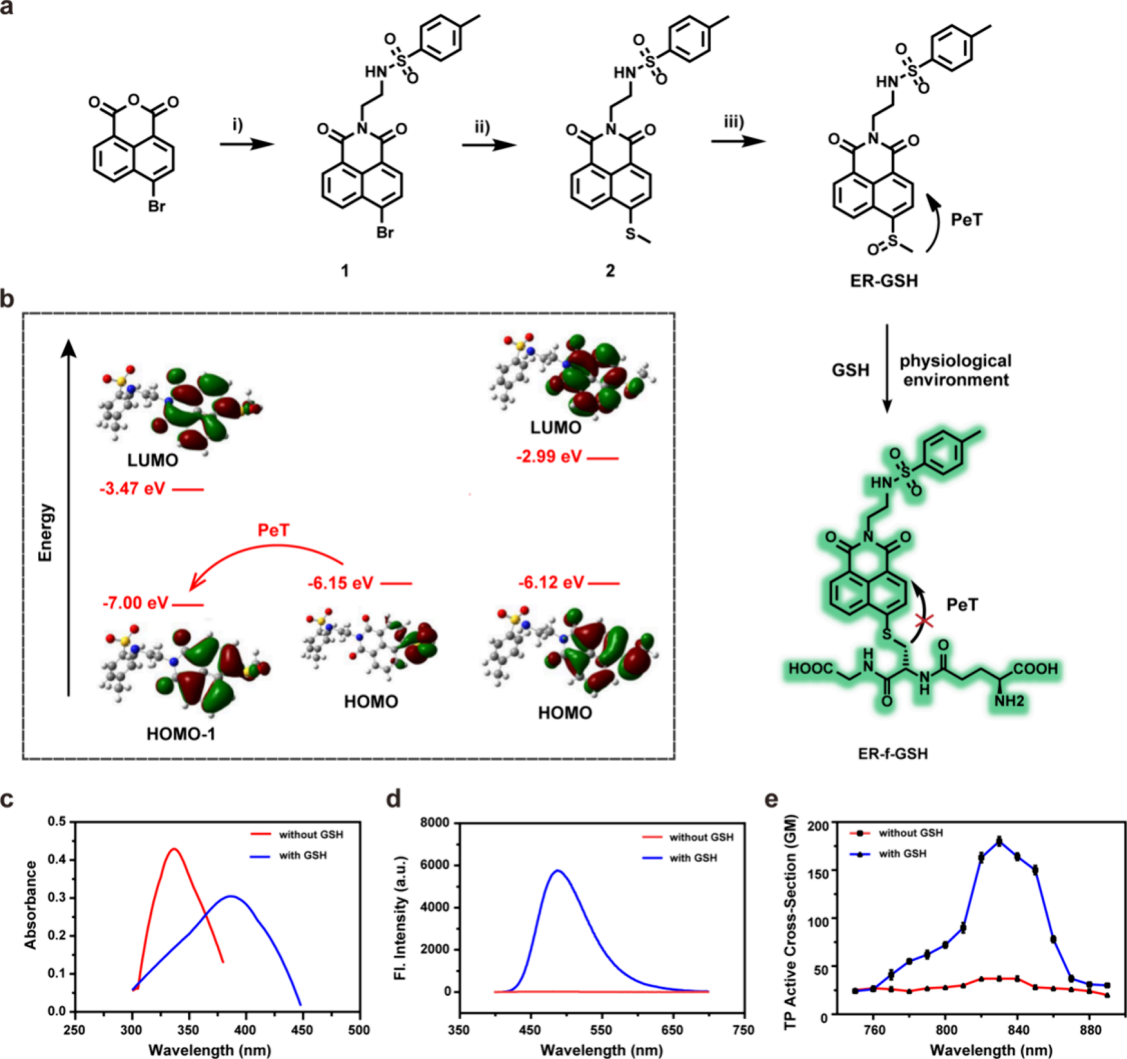
(a) Design, synthesis, and sensing mechanism of the probe ER-GSH. Reagents and conditions: (i) *N*-(2-aminoethyl)-4-methylbenzenesulfonamide, ethanol, 80 °C, 8 h. (ii) Sodium thiomethoxide, K_2_CO_3_, anhydrous DMF, 90 °C, 12 h. (iii) *m*-CPBA, anhydrous dichloromethane, 0 °C to room temperature, 2 h. (b) Optimized DFT molecular orbital plots (LUMO and HOMO) of ER-GSH (left) and ER-f-GSH (right) (c) absorption and (d) emission spectra of the probe ER-GSH before and after reaction with GSH. (e) Two-photon activity cross section of the probe ER-GSH before and after reaction with GSH.

To explore the response of ER-GSH to GSH, we used density functional theory (DFT) and time-dependent DFT (TDDFT) methods by employing a B3LYP hybrid functioal with the 6-31+G (d) basis set to calculate ER-GSH and its analogue, ER-f-GSH. Vibration frequency calculations were carried out to make sure that the optimized structures were true energy minima. All calculations were modeled by applying the self-consistent reaction field (SCRF) under the polarizable continuum model (PCM) incorporating water as the solvent and were carried out using the Gaussian 16 program.^19^ The frontier molecular orbital plots of ER-GSH and ER-f-GSH are given in Figure 1b. For ER-GSH, the highest occupied molecular orbital (HOMO) is situated on the methyl sulfoxide group, whereas the lowest unoccupied molecular orbital (LUMO) and HOMO-1 are located on the naphthalimide moiety. The transition of HOMO → LUMO, which is the main transition of the S_1_ state, is a full electron-transfer process (from the methyl sulfoxide group to the naphthalimide moiety); thus, there is no overlap between the HOMO and the LUMO. These results indicate that the S_1_ state is a dark state and that ER-GSH is probably nonfluorescent. The optimized geometric structure confirmed that the excitation wavelength of the S_0_ to S_2_ state with the highest oscillator strength was 340 nm (Table S1), thus correlating with an absorption of 346 nm in the UV–visible absorption spectrum. After the addition of GSH, the sulfoxide group is converted to ER-f-GSH. Significantly, both the HOMO and LUMO in ER-f-GSH are primarily situated on the naphthalimide group, thus preventing the PeT process and generating strong fluorescence in the naphthalimide. Calculations demonstrated that the emission wavelength was 454 nm (Table S2), which is consistent with experimental results at 488 nm. Furthermore, it should be noted that the methyl sulfide’s contribution to the HOMO and LUMO is different; it can be found that the electron densities of methyl sulfide decrease when transitioning from the HOMO to LUMO. This demonstrates that the charge is transferred from methyl sulfide to naphthalimide via the HOMO to LUMO transition process, indicating that the S_0_ to S_1_ state of ER-f-GSH has ICT characteristics. Our calculations demonstrate that ER-f-GSH undergoes a strong transition from S_0_ to S_1_ at 413 nm, with a maximum oscillator strength of 0.3335 (Table S1). This result agrees with the experimental value of 388 nm. These results rationalize the PeT and ICT mechanisms for ER-GSH and ER-f-GSH, therefore confirming our assumptions about the response mechanism of ER-GSH.

Figure 1c,d shows the spectral results for ER-GSH in PBS (10 mM containing 40% EtOH). Upon reaction with GSH, the PeT was removed and the ICT increased; thus, ER-GSH (10 μM) exhibited a remarkable red shift from 346 to 388 nm, and the fluorescence intensity of ER-GSH increased 304-fold at 488 nm. The two-photon active cross section (δΦ) of ER-f-GSH with added GSH, as can be seen in Figure 1e, is 180 GM at 830 nm, indicating that ER-GSH can use two-photon confocal imaging technology to analyze GSH concentrations. All compounds synthesized were characterized using ^1^H and ^13^C nuclear magnetic resonance spectroscopy, and the relevant data and experimental procedures are provided in the Supporting Information (Figures S1–S4).

### Spectroscopic Changes with the Addition of GSH

**Figure 2:**
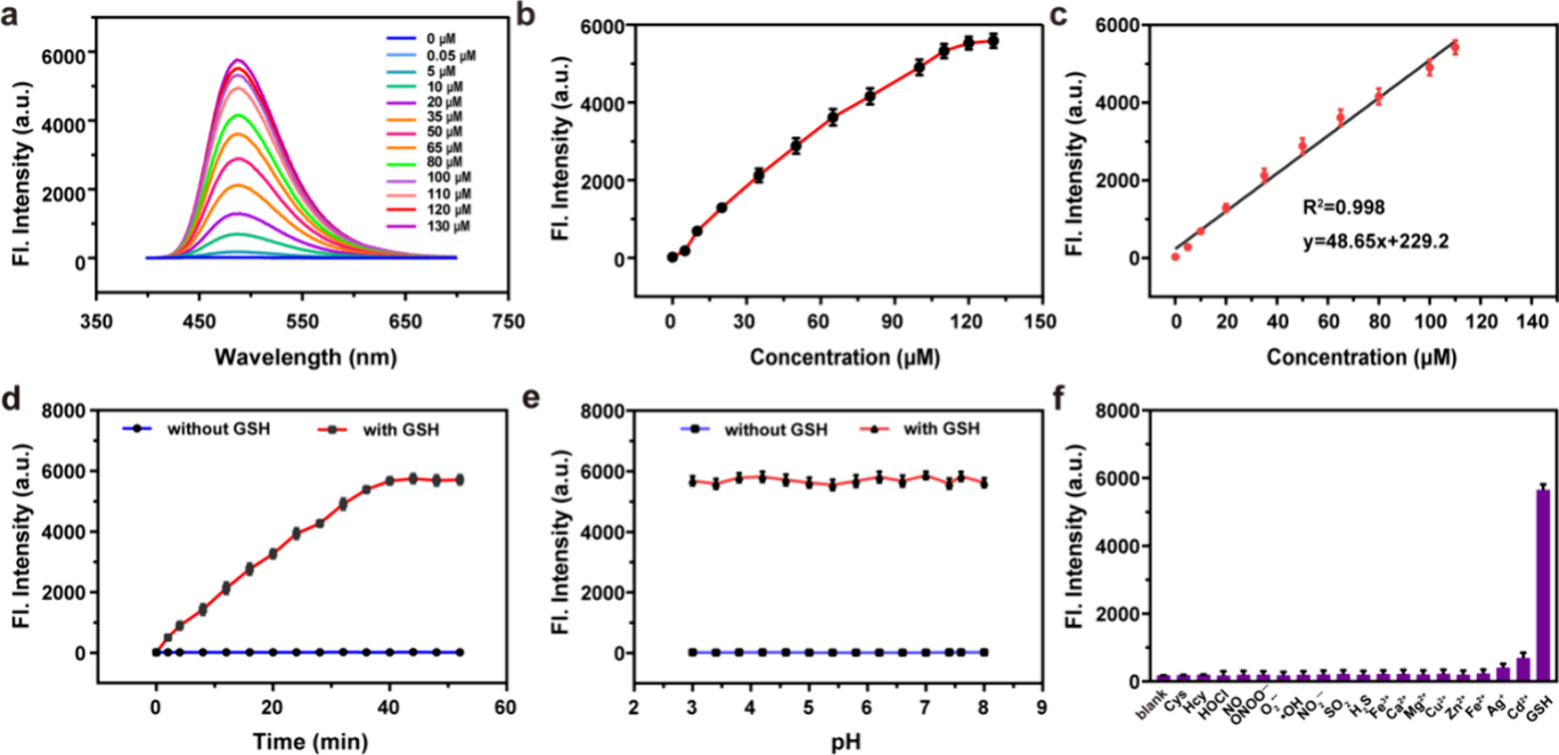
(a) Fluorescence spectra of ER-GSH (10 μM) with an increase in GSH concentration (0, 0.05, 5, 10, 20, 35, 50, 65, 80, 100, 110, 120, and 130 μM). (b) Calibration curve for the determination of GSH. (c) Liner relationship of fluorescence intensity with different concentrations of GSH. (d) Time-dependent emission of ER-GSH (10 μM) with and without GSH (110 μM). (e) Fluorescence emission of ER-GSH (10 μM) with or without GSH at different pH. (f) Fluorescence intensity of ER-GSH (10 μM) with 1.0 mM biomolecules (Cys and Hcy), 100 μM reactive oxygen species (·OH, O_2_^·–^, and HOCl), 100 μM active nitrogen (NO_2_^–^, ONOO^–^, and NO), 100 μM reactive sulfur species (SO_2_ and H_2_S), and 20 μM metal ions (Fe^2+^, Fe^3+^, Ca^2+^, Mg^2+^, Zn^2+^, Ag^+^, Cu^2+^, and Cd^2+^).

At first, we determined the solubility of ER- GSH at concentrations of 0.25, 1, 2, 5, 8, 10, 15, and 20 μM. The results revealed that even at a concentration of 20 μM, the solution had not attained saturation, confirming the suitability of ER-GSH for use in aqueous solutions and cell experiments (Figure S6). Subsequently, the concentration response of ER-GSH to GSH was investigated. As the GSH concentration increased, the fluorescence intensity of ER-GSH exhibited a linear increase from 0 to 110 μM at 488 nm (illustrated in Figure 2a,b). ER-GSH exhibited a detection limit of 37.8 nM for GSH (Figure 2c). At a GSH concentration of 110 μM, the fluorescence intensity of the reaction system reached a plateau within 40 min. Additionally, in the absence of GSH, the fluorescence intensity of the reaction solution remained constant (Figure 2d).

The effects of different pH on ER-GSH and its response to GSH were also determined by measuring the fluorescence intensity before and after reaction with GSH under various pH conditions (Figure 2e). The fluorescence intensity of ER-GSH remained constant over a pH range from 3.0 to 8.0, indicating that the reaction between ER-GSH and GSH is not sensitive to changes in pH, which confirms that ER-GSH is a suitable choice for the fluorescence imaging of GSH in living cells since the results would not be affected by changes in the intracellular pH. We then evaluated interferences for the reaction of ER-GSH with GSH, including 1.0 mM biomolecules (Cys and Hcy), 100 μM reactive oxygen species (·OH, O_2_·^–^, and HOCl), 100 μM active nitrogen (NO_2_^–^, ONOO^–^, and NO), 100 μM reactive sulfur species (SO_2_ and H_2_S), and 20 μM metal ions (Fe^2+^, Fe^3+^, Ca^2+^, Mg^2+^, Zn^2+^, Ag^+^, Cu^2+^, and Cd^2+^). Figure 2f confirms that these interferences did not lead to a significant fluorescence enhancement, whereas GSH caused a remarkable fluorescence enhancement. These results indicate that ER-GSH exhibits high selectivity toward GSH.

### Two-Photon Fluorescence Imaging of Cells

The toxicity of ER-GSH toward BV2 cells was determined to demonstrate its applicability for two-photon excitation mode live cell imaging. A 3-(4,5-dimethylthiazol-2-yl)-2,5-diphenyltetrazolium bromide (MTT) assay was employed to assess cytotoxicity in BV2 cells cultured at different concentrations (0–30 μM) of ER-GSH. As depicted in Figure S7, even at a concentration of 30 μM, ER-GSH exhibited low toxicity to cells, resulting in a 95% survival rate, rendering it suitable for imaging of living cells. Figure S8 illustrates the continuous excitation of ER-GSH with two photons at 830 nm for 60 min. The fluorescence intensity of ER-GSH, postreaction, remained relatively stable for 60 min, indicating its good photostability and providing an opportunity for extended cell imaging.

With demonstrated low toxicity and good photostability, ER-GSH could be used to detect both endogenous and exogenous GSH in cells. The fluorescence observed in the green channel (450–550 nm) of BV2 cells incubated with ER-GSH for 30 min, as shown in Figure S9, confirms the excellent cell permeability of ER-GSH and its ability to image endogenous GSH in cells. Initial treatment of BV2 cells with 30 μM *N*-ethylmaleimide (NEM) for 30 min, followed by a 30 min incubation with ER-GSH, rendered the fluorescence intensity of the green channel (*I*_green_) negligible. However, BV2 cells preincubated with NEM, followed by a 30 min incubation with ER-GSH and subsequent treatment with 500 μM GSH, Cys, and Hcy, exhibited a significant increase in cell *I*_green_ with only GSH. This suggests that ER-GSH is capable of detecting both endogenous and exogenous GSH.

Conducting an experiment to assess the intracellular distribution of ER-GSH allows us to determine its potential in specifically targeting ER stress. Co-staining ER-GSH with a commercially available red colocalization dye was used to explore the dye’s localizing efficiency within diverse subcellular organelles. Various red colocalization dyes, such as Lyso Tracker Red for lysosome, Mito Tracker Red for mitochondria, and ER Tracker Red for the endoplasmic reticulum (ER), were used alongside ER-GSH for a comprehensive examination of the probe’s subcellular targeting capabilities. To achieve this, BV2 cells loaded with the ER-GSH probe were incubated with 60 nM Lyso Tracker Red, Mito Tracker Red, and ER Tracker Red for 30 min, followed by confocal imaging. As illustrated in Figure S10, a pronounced overlap in fluorescence was observed between ER-GSH and ER Tracker Red, yielding a high Pearson coefficient of 0.93. However, in the case of the mitochondria and lysosomes, the Pearson coefficients were 0.44 and 0.31, respectively. This indicates that ER-GSH exhibits a specific affinity for the ER, thereby establishing a theoretical foundation for its utility in the *in situ* detection of cellular ER stress.

**Figure 3:**
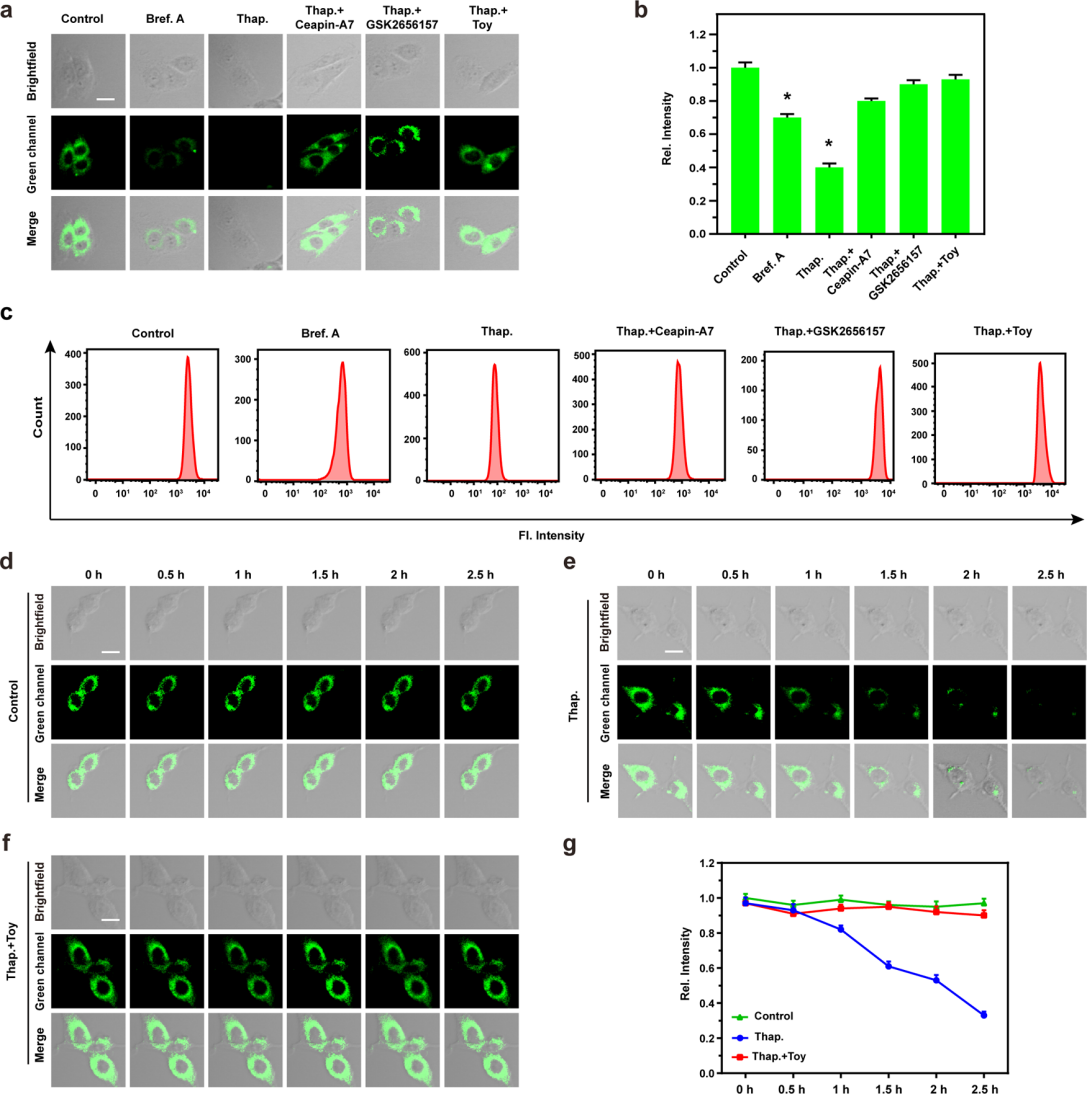
(a) Fluorescence confocal microscopic imaging of BV2 cells under different incubation conditions (Bref.A, Thap., Thap. + Ceapin-A7, Thap. + GSK2656157, Thap. + Toy). (b) Fluorescence intensity histogram of figure (a). The difference was analyzed by one-way ANOVA and Bonferroni post-test. *P < 0.05 vs control group. (c) Flow cytometry analysis after incubation under different conditions according to figure (a). (d–f) Fluorescence confocal microscopic imaging of BV2 cells incubated in different ways (control, Thap., Thap. + Toy) for a period of time (0–2.5 h). (g) Line diagram of fluorescence intensity during incubation according to (d–f). Scale bar: 50 μm.

To evaluate the efficacy of ER-GSH in monitoring ER stress, we employed a range of ER stress inducers, including Brefeldin A (Bref. A) and Thapsigargin (Thap.), as well as inhibitors such as Toyokamycin (Toy), Ceapin-A7, and GSK2656157.^20^ As illustrated in Figure 3, a notable decrease in the fluorescence intensity (*I*_green_) was observed in the Bref. A and Thap. groups compared to the Control group, indicating that Bref. A and Thap. induced ER stress, leading to GSH depletion in the cells. Conversely, the Thap. group exhibited a significant increase in *I*_green_ upon the addition of Ceapin-A7, GSK2656157, and Toy compared to the Bref. A and Thap. groups. This observation can be attributed to the ER stress inhibitory effects exerted by Ceapin-A7, GSK2656157, and Toy, resulting in a decrease in intracellular ER stress levels and subsequent elevation of GSH levels. Furthermore, comparison between the Thap. group and Thap. + Toy group revealed that the fluorescence intensity of the Thap. + Toy group, as well as the Control group, remained relatively stable within the 0–2.5 h time frame, whereas the Thap. group experienced a significant reduction in *I*_green_. These findings underscore the efficacy of ER-GSH as a precise ER-targeting tool, capable not only of detecting ER stress in cells but also of monitoring the ER stress process over an extended duration owing to its fluorescence stability.

### Scrap Leather-Induced ER Stress Model

Previous studies have highlighted the role of formaldehyde (FA) found in scrap leather in inflicting damage.^21−23^ In this study, we employed the ER stress inhibitor Toy and designed a biological model using free FA leather (nonformic acid tanning), FA leather (formic acid tanned and has undergone treatment to reduce emissions), and untreated FA leather (formic acid tanned and has not undergone treatment to reduce emissions), as depicted in Figure S11. Compared to the control group, a slight decrease in fluorescence intensity was observed in the Free FA leather group, whereas the decline was more pronounced in the FA leather group and Untreated FA leather group, with the latter exhibiting the lowest fluorescence intensity in *I*_green_. These experimental results suggest that the presence of FA in leather may lead to a reduction in intracellular GSH levels. However, pretreatment of cells with Toy resulted in no significant differences in fluorescence intensity regardless of the treatment applied, indicating that fluctuations in GSH levels induced by FA may be attributed to ER stress.

**Figure 4:**
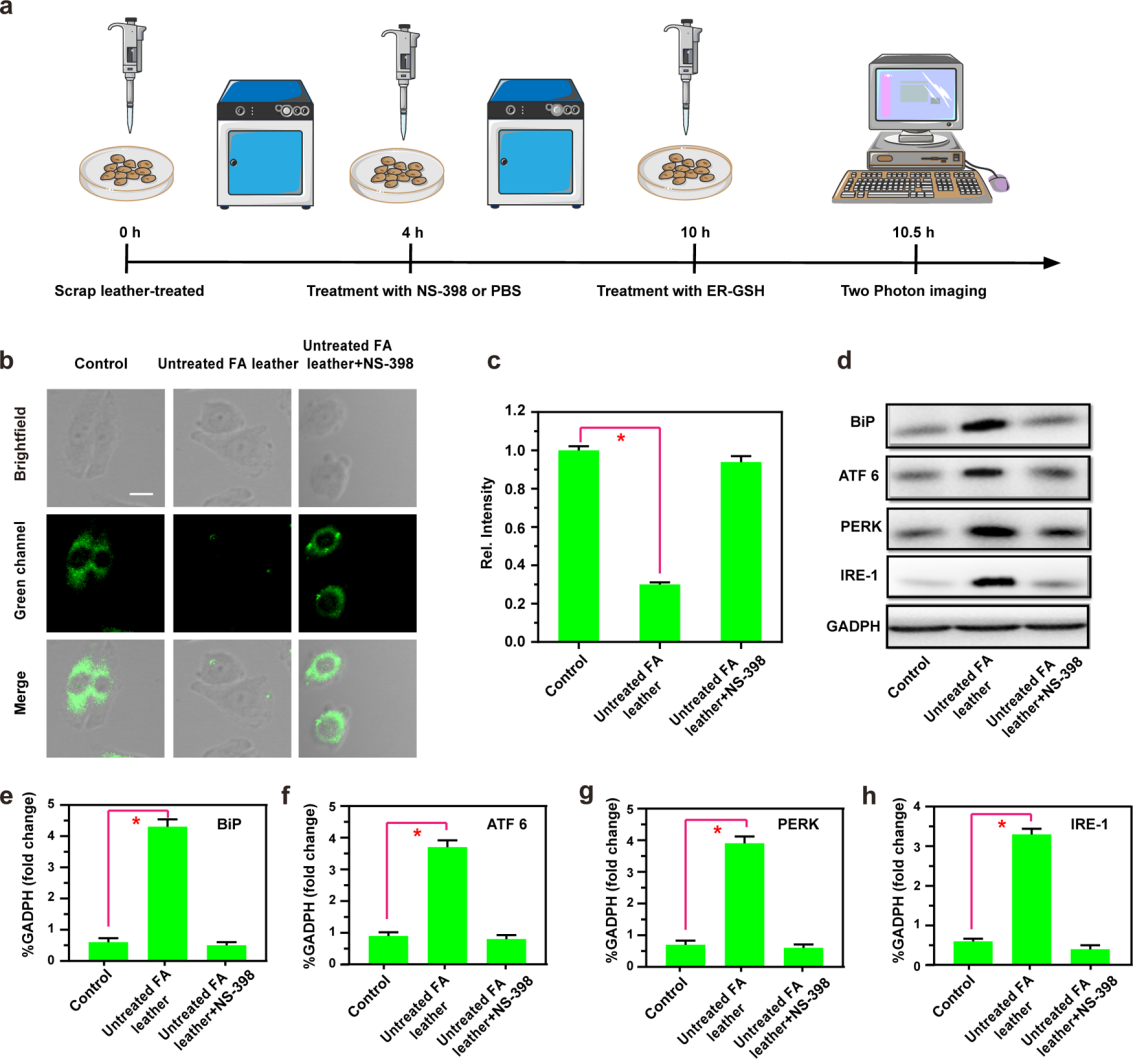
(a) Schematic illustration of the scrap leather and probe treatment. (b) Two-photon fluorescence imaging of BV2 cells after different treatments: Control group (untreated), Untreated FA leather (FA tanned leather without finishing treatment), Untreated FA leather + NS- 398 groups (FA tanned leather treated without finishing section was incubated with NS-398). (c) Histogram of the mean fluorescence intensity of (b); the data are expressed as mean ± SD, λ_ex_ = 830 nm, λ_em_ = 450–550 nm, scale bar: 50 μm. (d) Western blot of the expression of BiP, ATF6, PERK, IRE-1, and GAPDH in BV2 cells in (b). (e–h) is (d) quantitative statistics of protein expression. The difference was analyzed by one-way ANOVA and Bonferroni post-test. *P < 0.05 vs Control group.

Following this, our study investigated the mechanistic analysis of ER stress induction by Untreated FA leather, with results depicted in Figure 4a–c. As anticipated, compared to the control group, the *I*_green_ signal in the Untreated FA leather group was negligible, whereas pretreatment of cells with NS-398 (an anti-inflammatory drug)^24^ resulted in a significant increase in *I*_green_ signal. Western blotting was employed to analyze the expression of various ER stress-related proteins in the Untreated FA leather group and Untreated FA leather + NS-398 group. As depicted in Figure 4d–h, compared to the Control group, the Untreated FA leather group exhibited elevated levels of immunoglobulin binding proteins (BiP, a pivotal regulatory molecule of ER stress), protein kinase such as ER kinase (PERK, a marker for detecting ER protein misfolding), and ER stress proteins (ATF6, IRE-1, both crucial molecules in the three signaling pathways of ER stress).^25^ Upon administration of the anti-inflammatory drug NS-398, the Untreated FA leather + NS-398 group displayed a reduction in the content of associated proteins. These results indicate that NS-398 can effectively suppress the expression of ER stress-related proteins, suggesting its potential as a therapeutic agent for ER stress induced by leather products, possibly owing to its anti-inflammatory effects.

### Two-Photon Fluorescence Images of Mice Brain Tissue

**Figure 5:**
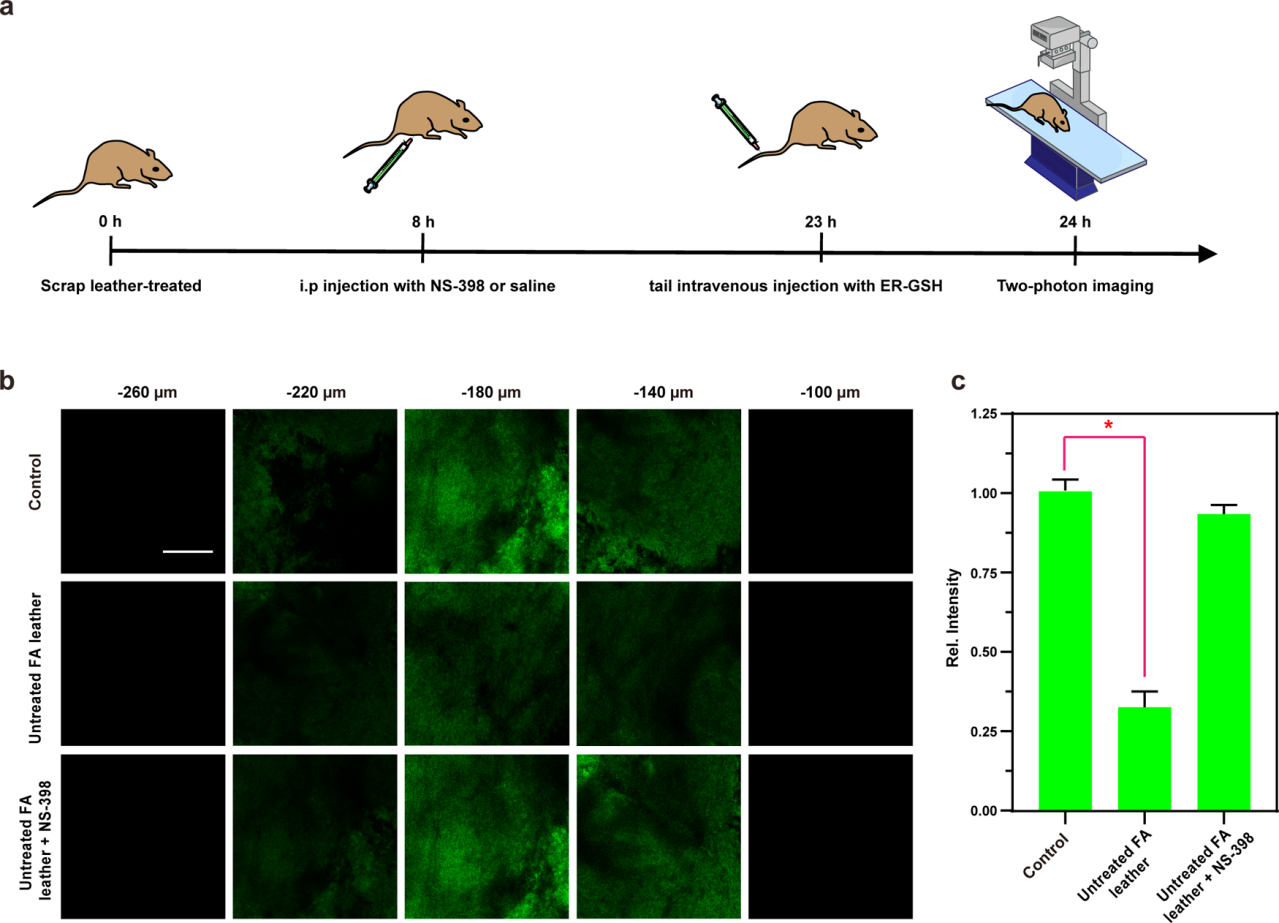
(a) Schematic illustration of scrap leather and probe treatment. (b) *In situ* TP fluorescence imaging of the brains of mice and (c) averaged fluorescence changes of ER-GSH loaded (100 μL, 200 μM) brains when subjected to different treatments: Sham group (untreated), Untreated FA leather treatment, and Untreated FA leather + NS-398 treatment. λ_ex_ = 830 nm; λ_em_ = 450–550 nm. Scale bar: 200 μm. The difference was analyzed by one-way ANOVA and Bonferroni post-test. *P < 0.05 vs Control group.

In preparation for applying ER-GSH to tissue imaging in living organisms, a comprehensive evaluation of its biocompatibility in various tissues is essential prior to mouse imaging experiments. Following the injection of the probe into the tail vein of mice, organs such as the brain, heart, liver, spleen, lung, and kidney were scrutinized using Hematoxylin and Eosin (H&E) staining. As depicted in Figure S12, ER-GSH did not induce significant tissue or organ damage, underscoring its biocompatibility for *in situ* imaging in mice. Consequently, ER-GSH holds promise for the subsequent experiments involving mouse tissue imaging. To investigate the ability of ER-GSH to monitor the ER stress process induced by scrap leather in the mouse brain (visual schematic for model construction shown in Figure 5a), with this research, we employed two-photon confocal Z-stack analysis and the results indicated that ER-GSH exhibited an impressive imaging depth within brain tissue, reaching depths of up to 220 μm (as depicted in Figure 5b). This observation underscores the effectiveness of two-photon confocal imaging in capturing ER-GSH dynamics. Subsequently, we delved into the fluorescence imaging capabilities of ER-GSH in mouse brain tissue. As evident in Figure 5c, the Untreated FA leather group exhibited a significant decrease in *I*_green_ compared to the Control group. This decline is attributed to leather products inducing ER stress in cells, depleting intracellular GSH, and resulting in a diminished fluorescence intensity of ER-GSH due to a reduction in the GSH content. Conversely, the *I*_green_ levels in the Untreated FA leather + NS-398 group was substantially higher than that of the Untreated FA leather group owing to the anti-inflammatory action of NS-398, which alleviated the ER stress in cells, thereby increasing the GSH content in brain tissue. This implies that NS-398 effectively impedes FA-induced ER stress in mouse brain tissue.

**Figure 6:**
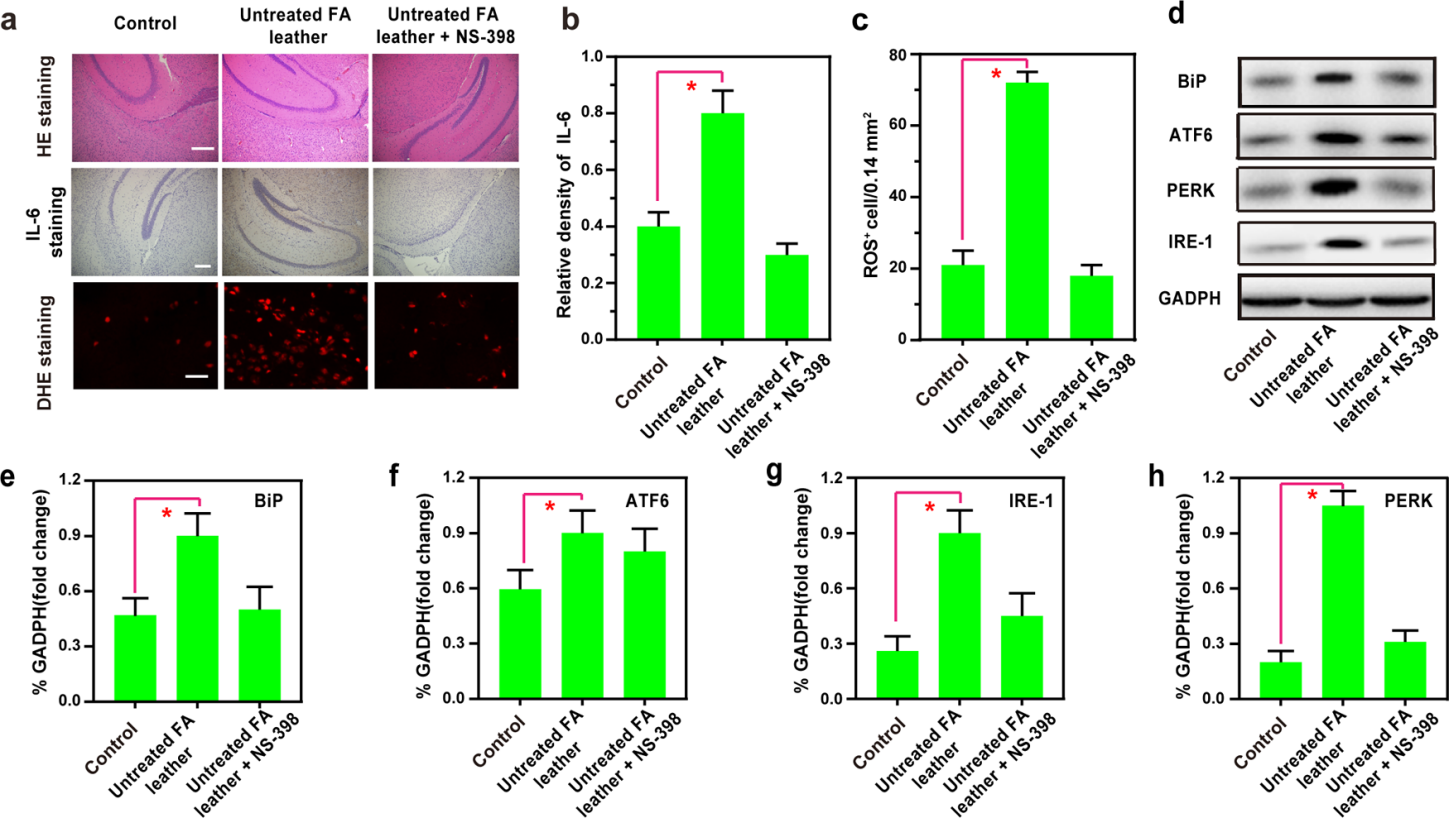
(a) Hematoxylin and eosin (H&E) staining (organ damage) analysis, IL-6 immunohistochemistry staining, immunofluorescent staining, and DHE staining of brain tissue when subjected to different treatments. (b, c) Quantification of IL-6 and ROS-positive cells from samples shown in (a). (d) Western blotting illustrating the expression of BiP, ATF6, IRE-1, and PERK from the samples in (a). Quantification data of the Western blot results of (e) BiP, (f) ATF6, (g) IRE-1, and (h) PERK from the samples in (d). Scale bars: 200 μm. The difference was analyzed by one-way ANOVA and Bonferroni post-test. *P < 0.05 vs Control group.

The H&E staining results vividly illustrated a discernible hippocampal structure of mice in the Control group, showcasing normal cell morphology. In contrast, the Untreated FA leather group exhibited an abnormal hippocampal tissue structure characterized by increased nuclear consolidation and cell count. Notably, the Untreated FA leather + NS-398 group displayed a mitigated effect, featuring less nuclear consolidation and a reduced presence of inflammatory cells (Figure 6a). The expression of IL-6 and ROS, as evidenced by dihydroethidium (DHE) staining, agreed with the immunohistochemistry staining results (Figure 6a–c). Furthermore, the data derived from the Western blotting analysis, exemplified in Figure 6d–h, substantiates the outcomes of the preceding investigations. The experiments revealed that the FA present in Untreated FA leather can induce ER stress in mouse brain cells. However, the administration of NS-398 exhibits a protective effect, shielding the mice from the harm caused by ER stress.

## Conclusions

With this research, we have evaluated the two-photon fluorescence probe ER-GSH, designed specifically for the turn-on detection of GSH. Composed of a naphthalimide fluorescent group, a GSH-triggered group, and an ER-targeting group (*p*-methylnenene sulfonamide), our probe exhibits rapid response, high selectivity, and sensitivity to GSH. The removal of the PeT effect, facilitated by the reduction of the sulfur–oxygen double bond, enhances the ICT effect, resulting in a remarkable 304-fold increase in fluorescence intensity and an impressive detection limit of 37.8 nM. Noteworthy for its low cytotoxicity and precise ER targeting, ER-GSH proves ideal for exploring the intricate relationship between living cells, ER stress induced by scrap leather in the mouse brain, GSH levels, and neuroinflammation. To the best of our knowledge, ER-GSH represents a pioneering two-photon fluorescence probe for the visualization and evaluation of cell ER stress triggered by scrap leather. Our research suggests that scrap leather may incite an inflammatory response within cells, culminating in diminished GSH levels and subsequent initiation of the ER stress pathway. Encouragingly, NS-398, an anti-inflammatory medication, can suppress cellular inflammation and ER stress progression by safeguarding cellular GSH levels. This investigation elucidates the intricate interplay between GSH and ER stress in neurons, offering profound implications for the early diagnosis of degenerative neural conditions.

## Supporting Information Available

Synthetic protocols; experimental methods; NMR and MS spectra; spectral analysis; and cellular and *in vivo* biosafety assessment and two-photon fluorescence imaging in tissues (PDF)

## References

[ref1] Kim J. S.; Sakamoto Y.; Takahashi F.; Shibata M.; Urano K.; Matsunaga S.; Yamaguchi-Shinozaki K.; Shinozaki K. Arabidopsis TBP-ASSOCIATED FACTOR 12 ortholog NOBIRO6 controls root elongation with unfolded protein response cofactor activity. Proc. Natl. Acad. Sci. U. S. A. 2022, 119 (6), e2120219119 10.1073/pnas.2120219119. 35115407 PMC8833210

[ref2] Janssen-Heininger Y.; Reynaert N. L.; van der Vliet A.; Anathy V. Endoplasmic reticulum stress and glutathione therapeutics in chronic lung diseases. Redox Biol. 2020, 33, 101516 10.1016/j.redox.2020.101516. 32249209 PMC7251249

[ref3] Wiseman R. L.; Mesgarzadeh J. S.; Hendershot L. M. Reshaping endoplasmic reticulum quality control through the unfolded protein response. Mol. Cell 2022, 82 (8), 1477–1491. 10.1016/j.molcel.2022.03.025. 35452616 PMC9038009

[ref4] Gardner B. M.; Pincus D.; Gotthardt K.; Gallagher C. M.; Walter P. Endoplasmic reticulum stress sensing in the unfolded protein response. Cold Spring Harbor Perspect. Biol. 2013, 5, a013169 10.1101/cshperspect.a013169. PMC3578356 23388626

[ref5] Song H.; Liu D.; Wang L.; Liu K.; Chen C.; Wang L.; Ren Y.; Ju B.; Zhong F.; Jiang X.; Wang G.; Chen Z. S.; Zou C. Methyltransferase like 7B is a potential therapeutic target for reversing EGFR-TKIs resistance in lung adenocarcinoma. Mol. Cancer 2022, 21, 43 10.1186/s12943-022-01519-7. 35144642 PMC8830004

[ref6] Yang Z.; Zou S.; Zhang Y.; Zhang J.; Zhang P.; Xiao L.; Xie Y.; Meng M.; Feng J.; Kang L.; Lee M. H.; Fang L. ACTL6A protects gastric cancer cells against ferroptosis through induction of glutathione synthesis. Nat. Commun. 2023, 14 (1), 4193 10.1038/s41467-023-39901-8. 37443154 PMC10345109

[ref7] Wang L.; Wang C. Oxidative protein folding fidelity and redoxtasis in the endoplasmic reticulum. Trends Biochem. Sci. 2023, 48 (1), 40–52. 10.1016/j.tibs.2022.06.011. 35871147

[ref8] Jakubek P.; Parchem K.; Wieckowski M. R.; Bartoszek A. The Interplay between Endogenous and Foodborne Pro-Oxidants and Antioxidants in Shaping Redox Homeostasis. Int. J. Mol. Sci. 2024, 25 (14), 7827 10.3390/ijms25147827. 39063068 PMC11276820

[ref9] Krawczyk M.; Burzynska-Pedziwiatr I.; Wozniak L. A.; Bukowiecka-Matusiak M. Impact of polyphenols on inflammatory and oxidative stress factors in diabetes mellitus: Nutritional antioxidants and their application in improving antidiabetic therapy. Biomolecules 2023, 13 (9), 1402 10.3390/biom13091402. 37759802 PMC10526737

[ref10] Wang P.; Yu L.; Gong J.; Xiong J.; Zi S.; Xie H.; Zhang F.; Mao Z.; Liu Z.; Kim J. S. An activity-based fluorescent probe for imaging fluctuations of peroxynitrite (ONOO^–^) in the alzheimer’s disease brain. Angew. Chem., Int. Ed. 2022, 61, e202206894 10.1002/anie.202206894. 35789171

[ref11] Han X.; Xing Y.; Song X.; Dou K.; Yu F.; Chen L. Bioimaging of glutathione variation for early diagnosis of hepatocellular carcinoma using a liver-targeting ratiometric near-infrared fluorescent probe. J. Mater. Chem. B 2023, 11, 6612–6620. 10.1039/D3TB00893B. 37357637

[ref12] He R.; Tang D.; Xu N.; Liu H.; Dou K.; Zhou X.; Yu F. Evaluation of erastin synergized cisplatin anti-nasopharyngeal carcinoma effect with a glutathione-activated near-infrared fluorescent probe. Chin. Chem. Lett. 2024, 35, 108658 10.1016/j.cclet.2023.108658.

[ref13] Xing P.; Niu Y.; Mu R.; Wang Z.; Xie D.; Li H.; Dong L.; Wang C. A pocket-escaping design to prevent the common interference with near-infrared fluorescent probes in vivo. Nat. Commun. 2020, 11 (1), 1573 10.1038/s41467-020-15323-8. 32218438 PMC7099068

[ref14] Wu X.; Wang R.; Qi S.; Kwon N.; Han J.; Kim H.; Li H.; Yu F.; Yoon J. Rational design of a highly selective near-infrared two-photon fluorogenic probe for imaging orthotopic hepatocellular carcinoma chemotherapy. Angew. Chem., Int. Ed. 2021, 60 (28), 15418–15425. 10.1002/anie.202101190. 33942436

[ref15] Zou Y.; Li M.; Xing Y.; Duan T.; Zhou X.; Yu F. Bioimaging of glutathione with a two-photon fluorescent probe and its potential application for surgery guide in laryngeal cancer. ACS Sensors 2020, 5, 242–249. 10.1021/acssensors.9b02118. 31815435

[ref16] Gupta A.; Khammash M. Frequency spectra and the color of cellular noise. Nat. Commun. 2022, 13 (1), 4305 10.1038/s41467-022-31263-x. 35879291 PMC9314361

[ref17] Cheng F.; Qiang T.; Wang B.; Ren L.; Hu W.; James T. D. Observation of formaldehyde-induced ER stress by an ER-targeting two-photon probe. Sens. Actuators B-Chem. 2024, 401, 134915 10.1016/j.snb.2023.134915.

[ref18] Jiang Y. J.; Cheng J.; Yang C. Y.; Hu Y. Z.; Li J.; Han Y. F.; Zang Y.; Li X. An ultrasensitive fluorogenic probe for revealing the role of glutathione in chemotherapy resistance. Chem. Sci. 2017, 8, 8012–8018. 10.1039/C7SC03338A. 29568448 PMC5853925

[ref19] Zhang J.; Alam P.; Zhang S.; Shen H.; Hu L.; Sung H. H. Y.; Williams I. D.; Sun J.; Lam J. W. Y.; Zhang H.; Tang B. Z. Secondary through-space interactions facilitated single-molecule white-light emission from clusteroluminogens. Nat. Commun. 2022, 13 (1), 3492 10.1038/s41467-022-31184-9. 35715394 PMC9205862

[ref20] Verwilst P.; Kim K.; Sunwoo K.; Kim H. R.; Kang C.; Kim J. S. Revealing Protein Aggregates under Thapsigargin-Induced ER Stress Using an ER-Targeted Thioflavin. ACS Sens. 2019, 4, 2858–2863. 10.1021/acssensors.9b00568. 31617349

[ref21] Ding W.; Wang Y.; Sun J.; Bao L.; Pang X. Dialdehyde sodium alginate bonded Dicyandiamide for formaldehyde-free leather production with enhanced properties. Carbohyd. Polym. 2022, 295, 119838 10.1016/j.carbpol.2022.119838. 35989032

[ref22] Xu S. L.; Yan K. C.; Xu Z. H.; Wang Y.; James T. D. Fluorescent probes for targeting the Golgi apparatus: design strategies and applications. Chem. Soc. Rev. 2024, 53, 7590–7631. 10.1039/D3CS00171G. 38904177

[ref23] Liang X.-G.; Cheng J.; Qin S.; Shao L.-X.; Huang M.-Z.; Wang G.; Han Y.; Han F.; Li X. Conformational restraint as a strategy for navigating towards lysosomes. Chem. Commun. 2018, 54, 12010–12013. 10.1039/C8CC06155F. 30204171

[ref24] Ho Y.; Li Z. L.; Shih Y. J.; Chen Y. R.; Wang K.; Whang-Peng J.; Lin H. Y.; Davis P. J. Integrin αvβ3 in the Mediating Effects of Dihydrotestosterone and Resveratrol on Breast Cancer Cell Proliferation. Int. J. Mol. Sci. 2020, 21 (8), 2906 10.3390/ijms21082906. 32326308 PMC7216104

[ref25] Ru Q.; Li Y.; Xie W.; Ding Y.; Chen L.; Xu G.; Wu Y.; Wang F. Fighting age-related orthopedic diseases: focusing on ferroptosis. Bone Res. 2023, 11 (1), 12 10.1038/s41413-023-00247-y. 36854703 PMC9975200

