# Supplementary material for: Monitoring Glutathione Content of the Endoplasmic Reticulum under Scrap Leather-Induced Endoplasmic Reticulum Stress via an Endoplasmic Reticulum-Targeted Two-Photon Fluorescent Probe
Source: Anal Chem. 2024 Oct 30;96(45):18132–40. doi: 10.1021/acs.analchem.4c04157 (PMC11561880; doi:10.1021/acs.analchem.4c04157)
Supplement: Supplementary file 1 — ac4c04157_si_001.pdf [file ac4c04157_si_001.pdf]

Supplementary Information

Monitoring Glutathione Content of the Endoplasmic Reticulum under Scrap Leather-Induced Endoplasmic Reticulum Stress via an Endoplasmic Reticulum-Targeted Two-photon Fluorescent Probe

Xinjian Song[a, *], Xumei Wang[a], Yiqian Hao[b], Chenchen Li[a], Li Chai[b], Haixian Ren[b, *], Jianbin Chen[e], Wei Hu[a, b, *] and Tony D. James[c, d, *]

[a] Hubei Key Laboratory of Biological Resources Protection and Utilization, School of Chemical and Environmental Engineering, Hubei Minzu University, Enshi, Hubei 445000, China.

[b] Department of Chemistry, Xinzhou Normal University, Xinzhou, Shanxi 034000, China.

[c] Department of Chemistry, University of Bath, Bath BA27AY, United Kingdoms; E-mail: t.d.james@bath.ac.uk.

[d] School of Chemistry and Chemical Engineering, Henan Normal University, Xinxiang, Henan 453007, China.

[e] School of Chemistry and Chemical Engineering, Qilu University of Technology (Shandong Academy of Sciences), Jinan, Shandong 250353, China.

Table of contents

GENERAL INFORMATION ON MATERIALS AND METHODS S3
SYNTHETIC ROUTE TO ER-GSH S4
FLUORESCENT AND UV-VIS ANALYSIS S5
Spectroscopic measurements S5
Determination of the detection limit S5
Quantum yield measurements S5
Measurement of two-photon cross section S6
Water solubility assay. S6
BIOLOGICAL ANALYSIS S8
Cell culture and imaging S8
Cytotoxicity assay S8
Western blotting assay S8
Histological staining of the tissue slices S9
Calculation of mean fluorescence intensity S9
Immunofluorescence staining S10
Two-photon fluorescence imaging in tissue S10
Measurement of ROS S11
SUPPLEMENTARY FIGURES AND TABLES S12
REFERENCES S26

GENERAL INFORMATION ON MATERIALS AND METHODS

Unless otherwise stated, all solvents and reagents were purchased from commercial suppliers and were used as received without further purification. BV2 cells were obtained from Procell Life Science & Technology Co., Ltd. 3-(4,5-Dimethylthiazol-2-yl)-2,5-diphenyltetrazolium bromide (MTT), Lyso Tracker Red, Mito Tracker Red, ER Tracker Red, GSH, Cys, Hcy, N-ethyl maleimide (NEM), Brefeldin A (Bref. A), Thapsigargin (Thap.), Toyokamycin, Ceapin-A7, GSK2656157, DTT, NS-398, FA, Bip, PERK, ATF6 and IRE-1 were purchased from Sigma-Aldrich. All aqueous solutions were prepared in ultrapure water with a resistivity of 18.25 MΩ cm (purified by Milli-Q system, Millipore). High-resolution mass spectrometry was performed with LTQ FT Ultra (Thermo Fisher Scientific, America) in MALDI-DHB mode. NMR spectra were recorded on a Bruker AVANCE III 500 spectrometer, using TMS as an internal standard. Two-photon microscopy (TPM) images were performed on a confocal laser scanning microscope (CLSM, C1-Si, Nikon, Japan).

SYNTHETIC ROUTE TO ER-GSH

ER-GSH was synthesized according to the procedure shown in Figure 1a, Compound 1 was synthesized in accordance with previously reported studies[1], and detailed as following:

Synthesis of compound 2: Compound 1 (300 mg, 0.63 mmol) was dissolved in 5 mL of DMF. Anhydrous potassium carbonate (174 mg, 1.26 mmol) and sodium methanethiolate (88 mg, 0.126 mmol) were added to the solution under nitrogen protection. The reaction mixture was heated at 90 °C for 4 h. After completion of the reaction, the mixture was cooled and poured into ice water, resulting in the precipitation of a solid. Compound 2 (207 mg, yellow crystals) was obtained with a yield of 74%. ^{1}H NMR (500 MHz, DMSO-d_6) δ 8.46 (dd, J = 8.1, 1.3 Hz, 1H), 8.38 (d, J = 7.7 Hz, 2H), 7.83 (dd, J = 8.2, 7.6 Hz, 1H), 7.73 – 7.67 (m, 2H), 7.64 (t, J = 6.4 Hz, 1H), 7.62 – 7.57 (m, 1H), 7.38 – 7.32 (m, 2H), 4.16 (dt, J = 12.3, 6.2 Hz, 2H), 3.20 (q, J = 6.3 Hz, 2H), 2.50 (s, 3H), 2.39 (s, 3H).

Synthesis of compound ER-GSH: Compound 2 (200 mg, 0.45 mmol) was dissolved in 15 mL of dichloromethane at 0 °C, and stirred for 10 min. Then, a solution of meta-chloroperoxybenzoic acid (m-CPBA, 78 mg, 0.45 mmol) in 5 mL of dichloromethane was slowly added dropwise while maintaining the temperature at 0 °C. The reaction mixture was further stirred at 0 °C for 15 min and then gradually raised to room temperature while stirring for an additional 2 h. After completion of the reaction, the mixture was directly rotary-evaporated to separate the product. A yield of 161.7 mg of **ER-GSH** (78%) was obtained. ^{1}H NMR (500 MHz, DMSO-d_6) δ 8.67 (dd, J = 7.5, 1.3 Hz, 1H), 8.51 – 8.42 (m, 2H), 8.20 (d, J = 8.8 Hz, 1H), 7.91 (t, J = 7.9 Hz, 1H), 7.73 – 7.67 (m, 2H), 7.64 (t, J = 6.4 Hz, 1H), 7.38 – 7.32 (m, 2H), 4.16 (dt, J = 12.3, 6.2 Hz, 2H), 3.20 (q, J = 6.3 Hz, 2H), 2.74 (s, 3H), 2.39 (s, 3H). ^{13}C NMR (125 MHz, DMSO-d_6) δ 164.42, 164.34, 145.91, 142.38, 137.70, 130.57, 129.57, 129.55, 127.81, 127.60, 127.31, 127.25, 127.21, 126.78, 125.58, 124.44, 42.00, 41.91, 41.37, 21.50. HR-MS (MALDI-DHB) calcd for $C_{22}H_{20}N_2O_5S_2$ $[M+H]^+$: 457.0886, found: 457.0834.

FLUORESCENT AND UV-VIS ANALYSIS

Spectroscopic measurements

Unless otherwise mentioned, all the measurements using ER-GSH were tested in PBS buffer (10 mM, containing 40% EtOH) system. The excitation wavelength was 388 nm under one-photon mode. After adding GSH and incubating at 37 °C for 5 min in a thermostat, a 500 μL aliquot of the reaction solution was transferred to a quartz cell with an optical length of 1 cm for the measurement of absorbance or fluorescence. The excitation wavelength was 830 nm, under two-photon excitation mode.

For the selectivity assay, •OH was generated by Fenton reaction between Fe^{3+} (EDTA) and H_2O_2 quantitatively, and Fe^{2+} (EDTA) concentrations represented •OH concentrations[2]. The $ONOO^-$ source was the donor 3-morpholinosydnonimine hydrochloride (SIN-1)[3]. NO was generated in form of 3-(aminopropyl)-1-hydroxy-3-isopropyl-2-oxo-1-triazene (NOC-5)[4]. H_2O_2 was determined at 240 nm ($\varepsilon_{240\ nm}$ = 43.6 $M^{-1}cm^{-1}$). NO_2^- was generated from $NaNO_2$. All the reagents were obtained from Aladdin (USA). All other chemicals were from commercial sources and of analytical reagent grade unless indicated otherwise.

Determination of the detection limit

The limit of detection (LOD) for hypochlorous acid was calculated based on the following equation[5]:

$$LOD = 3\sigma/k$$

Where σ represents the standard deviation and k represents the slope of the titration spectra curve among the limited range.

Quantum yield measurements

The measurement of the fluorescence quantum yield was measured by using an

ethanol solution of rhodamine B as a standard (10 μM, $\Phi_r = 0.71$) and using the following equation[6].

$$\Phi_s = (A_r \cdot F_S \cdot n_s^2)/(A_s \cdot F_r \cdot n_r^2) \cdot \Phi_r \ (A \leq 0.05)$$

Where s and r represent the sample to be tested and the reference dye, respectively. A represents the absorbance at the maximum absorption wavelength, F represents the fluorescence spectrum integral at the maximum absorption wavelength excitation, and n represents the refractive index of the sample to be tested or the reference dye solvent.

Measurement of two-photon cross section

The two-photon cross section (δ) was determined by using reported femto second (fs) fluorescence measurement techniques[7]. ER-GSH was dissolved in PBS buffer or glycerol and the two-photon induced fluorescence intensity was measured at 750–880 nm by using rhodamine B as the reference, whose two-photon property has been well characterized in the literature. The intensities of the two-photon induced fluorescence spectra of the reference and sample emitted at the same excitation wavelength were determined. The two-photon cross section was calculated by using the following equation.

$$\delta_s = \delta_r \cdot (S_s \cdot \Phi_r \cdot \varphi_r \cdot c_r)/(S_r \cdot \Phi_s \cdot \varphi_s \cdot c_s)$$

Where the subscripts s and r stand for the sample and reference molecules, respectively. The intensity of the signal collected by a CCD detector was denoted as S. Φ is the fluorescence quantum yield. is the overall fluorescence collection efficiency of the experimental apparatus which can be approximated by refractive index of the solvent. The number density of the molecules in solution was denoted as c. δ_r is the two-photon absorption cross section of the reference molecule.

Water solubility assay

A small amount of ER-GSH was dissolved in DMSO to prepare the stock solutions (0.01 M). In all cases, the concentration of EtOH was maintained to be 40%. The plot

of fluorescence intensity against the ER-GSH concentration was linear at low concentrations and exhibited downward curvature at higher concentrations, and the maximum concentration in the linear region was taken as the solubility.

BIOLOGICAL ANALYSIS

Cell culture and imaging

BV2 were cultured in Dulbecco's modified Eagle's medium (DMEM, Thermo Scientific) supplemented with 1% penicillin/streptomycin and 10% fetal bovine serum (FBS), and incubated in an atmosphere of 5/95 (v/v) of CO_2/air at 37 °C. Two days before imaging, the cells were passed and placed into glass-bottomed dishes (NEST). For labeling, the cells were washed with serum-free DMEM and then incubated with 5 μM ER-GSH (containing 1% DMSO) for 30 min at 37 °C.

Cytotoxicity assay

MTT tests were performed with reference to the reported protocol[8] with a minor change. BV2 cells were seeded in 96-well plates and incubated with different concentrations of ER-GSH (0, 10, 20, 30 and 40 μM, containing 1% DMSO in 200 μL DMEM). The experiment and control groups were incubated in an atmosphere of 5/95 (v/v) of CO_2/air at 37 °C for 24 h. Next, 20 μL 5.0 mg/mL MTT solution was added into each well, followed by incubation for 4 h under the same conditions. Then the 100 μL supernatant were removed and 150 μL DMSO added. After shaking for 10 min, the absorbance at 490 nm was measured by microplate reader (Synergy 2, BioTek Instruments Inc.). Cell survival rate was calculated by $A/A_0 \times 100\%$ (A and A_0 are the absorbance of the experimental group and control group, respectively).

Western blotting assay

Western blotting was carried out as previously described[9]. Cortical sections 1.0 to 2.0 mm from ipsilateral brain tissue was harvested and homogenized in cold RIPA buffer (C1053, Applygen, Beijing, China) plus protease inhibitor cocktail (G2006, Servicebio, Wuhan, China). The homogenates were centrifuged at 4 °C at 10, 000 × g for 30 min, and then the supernatants were harvested. Protein content was determined with the BCA kit (G2026, Servicebio, Wuhan, China). Protein samples (20 μL/lane)

were separated by electrophoresis on 4–15% sodium dodecyl sulfate-polyacrylamide gels and then transferred onto PVDF membranes (Millipore, Billerica, MA, USA). The corresponding membranes were cut according to the molecular weight of the protein to be detected, and blocked in 5% BSA for 1 h at room temperature. After incubation with the corresponding primary antibody overnight at 4 °C, the membranes were washed three times with TBST and then incubated with the secondary antibody (Cell Signaling Technology) of the corresponding species for 1h at room temperature. Finally, the membranes were incubated with ECL solution and the signal was detected using the imaging system from (LI-COR, Lincoln, NE, USA). The relative band intensity was calculated using Quantity One v4.6.2 software (Bio-Rad Laboratories, Hercules, USA) and then normalized to the GADPH loading control. All above experiments were repeated three times.

The following primary antibodies are used: anti-BiP (1:1,000; 63411, Cell Signaling Technology, Boston, USA), anti-ATF6 (1:1,000; 65880, Cell Signaling Technology, Boston, USA), anti-PERK (1:1,000; 5683, Cell Signaling Technology, Boston, USA) and anti-IRE-1 (1:1,000; 3294, Cell Signaling Technology, Boston, USA).

Histological staining of the tissue slices

After imaging, the mice were killed, and the livers were collected for tissue analysis. Through a series of standard procedures, including fixation in 10% neutral buffered formalin, embedding into paraffin and sectioning at 3 μm thickness, the tissues were stained with hematoxylin-eosin (H&E). Thereafter, the prepared slices were examined using a digital microscope.

Calculation of mean fluorescence intensity

The mean fluorescence density was measured by Image-Pro Plus (v. 6.0) and calculated via the equation (mean density = $IOD_{sum}/area_{sum}$), where IOD and area were integral optical density and area of fluorescent region.

Immunofluorescence staining

Scrap leather-operated mice were euthanized and perfused with cold PBS, followed by fixation with 4% paraformaldehyde for 2 days. The ischemic brains were cut into 50-μm sections and the free-floating slices were blocked with 0.1 M PBS containing 5% fetal bovine serum and 0.3% Triton X for 1 h at room temperature. After washing, the slices were incubated at 4 °C overnight with the following primary antibodies: anti-IL-6 (1:200; 12912, Abcam, Cambridge, England). The slices were then rinsed anti- and incubated with an Alexa 594-conjugated antibody (1:200; ANT030, Millipore, Billerica, MA) or an Alexa 488-conjugated antibody (1:200; ANT024, Millipore, Billerica, MA for 2 h at room temperature. After thorough rinsing, the nuclei were stained with DAPI (94010, Vector Laboratories, Burlingame, CA, USA). All slices were photographed using a confocal fluorescence microscope (BX63, Olympus Optical Ltd, Tokyo, Japan). The number of immunoreactive cells in predefined areas were quantified using ImageJ software (Media Cybernetics Inc., Rockville, MD, USA). Six different fields for each mouse and six mice for each group were counted. All counts were conducted by blinded observers.

Two-photon fluorescence imaging in tissue

All animal procedures were performed in accordance with the Guidelines for Care and Use of Laboratory Animals of Wuhan University and experiments were approved by the Animal Ethics Committee of College of Biology (Wuhan University). Wild-type C57BL/6J mice (n = 3; 25–30 g) were purchased from Hubei Experimental Animal Research Center. (Hubei, China; No. 43004700018817, 43004700020932). All animal experimental protocols were approved by the Animal Experimentation Ethics Committee of Wuhan University (No. WDRM-20170504) and were conducted according to the Animal Care and Use Committee guidelines of Renmin Hospital of Wuhan University. Animals were housed in a room with controlled humidity (65 ± 5%) and temperature (25 ± 1 °C), under a 12/12-hour light/dark cycle with free access to food and water for at least 1 week before the experiments. After the Scrap leather-

operated (cut the scrap leather into small pieces and wrap them around the mice. The mice were exposed to these leather pieces directly, and allowed to cohabitate in a relatively enclosed environment) model was successfully established, ER-GSH (100 μL, 200 μM) was injected through the tail vein, and the mice were anesthetized and dissected to remove the mouse brain tissue, and a 300-μm section was prepared with a microtome. Two-photon excited tissue fluorescence images were obtained by Zeiss LSM 710 multiphoton laser scanning confocal microscope.

Measurement of ROS

To assess ROS production, the brain was carefully and quickly isolated and cut into 4.0-μm sections and placed on chilled microscope slides. The samples were incubated in physiological saline solution containing 10 μM dihydroethidium (DHE; Sigma-Aldrich) for 30 min at 37 °C in the dark room. The brain was washed twice with PSS and placed under automatic fluorescence microscope (BX63, Olympus Optical Ltd, Tokyo, Japan).

SUPPLEMENTARY FIGURES AND TABLES

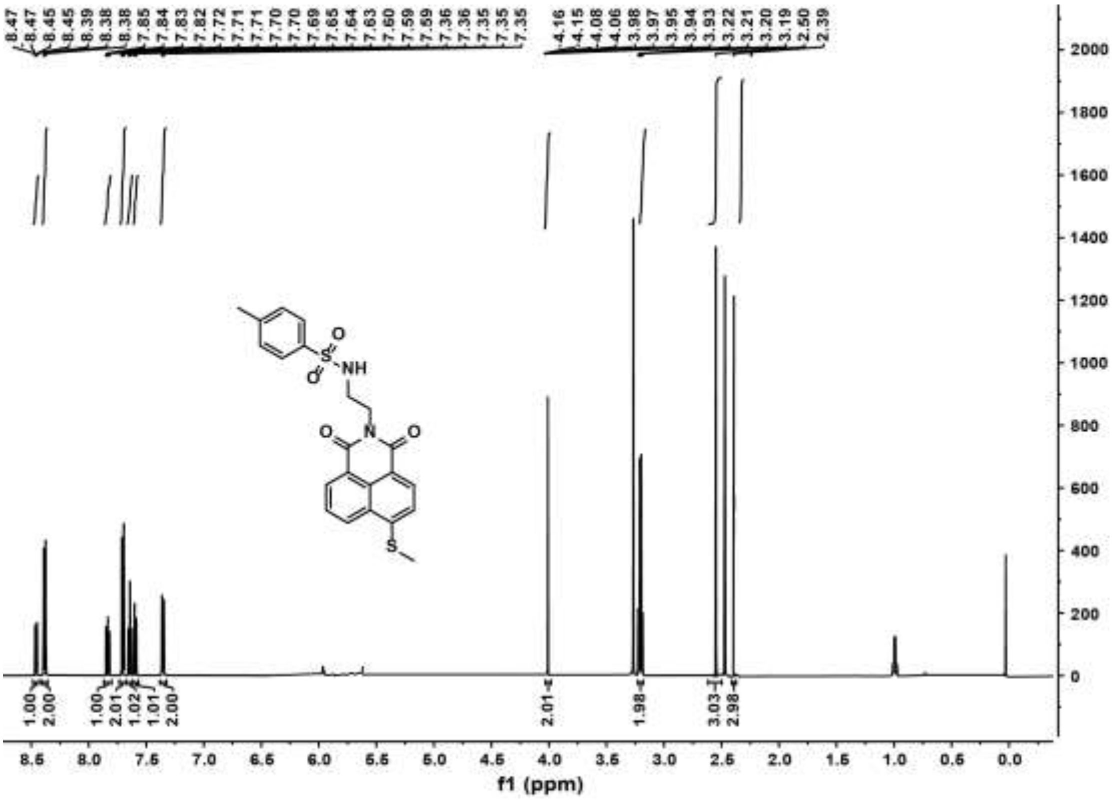

Figure S1. ^{1}H NMR spectrum (DMSO-d_6) of compound **2**.

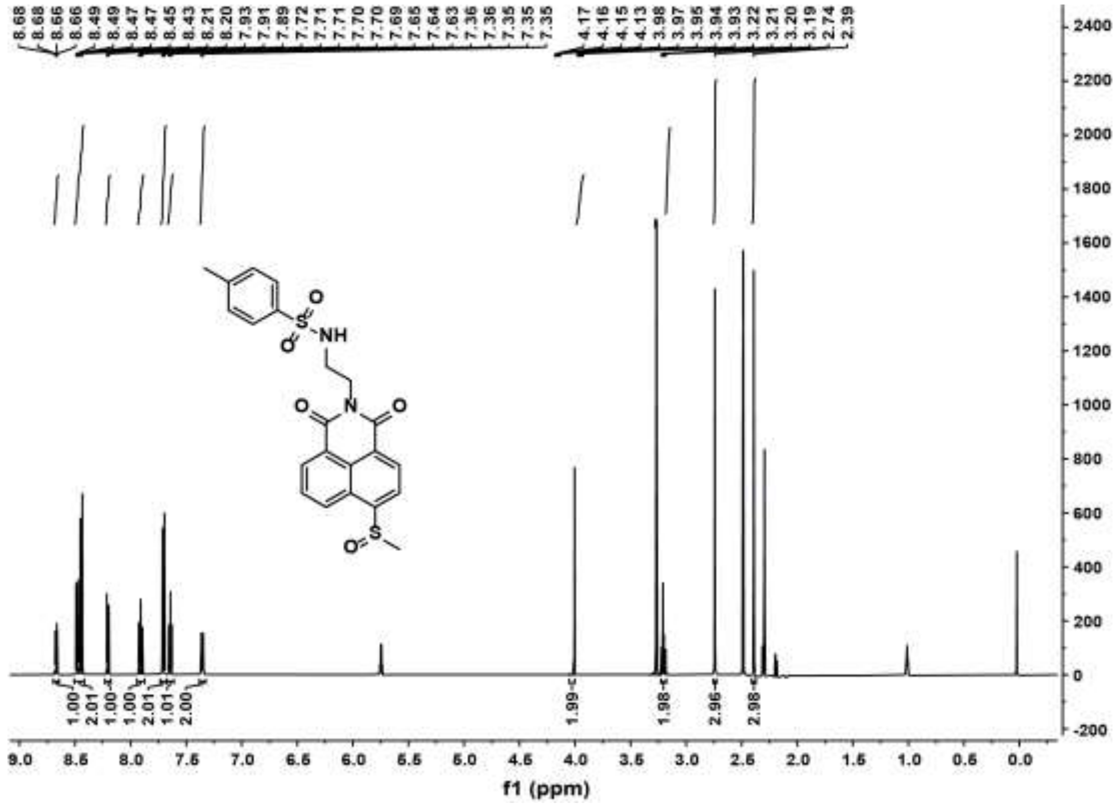

Figure S2. ^{1}H NMR spectrum (DMSO-d_6) of ER-GSH.

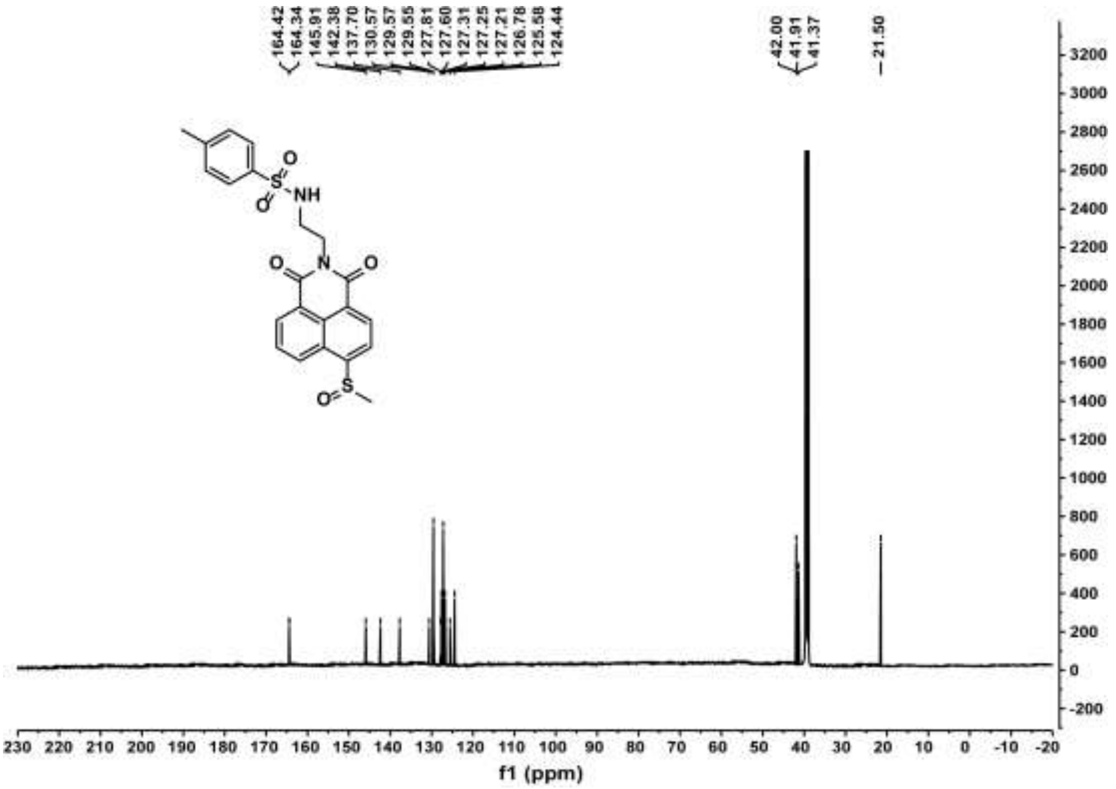

Figure S3. ^{13}C NMR spectrum (DMSO-d_6) of ER-GSH.

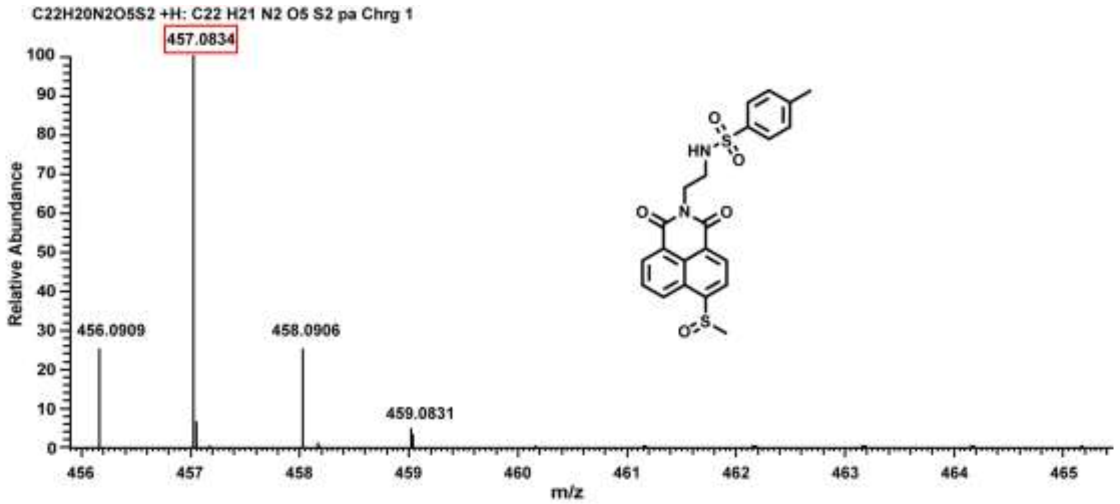

Figure S4. HR-MS spectrum of probe ER-GSH.

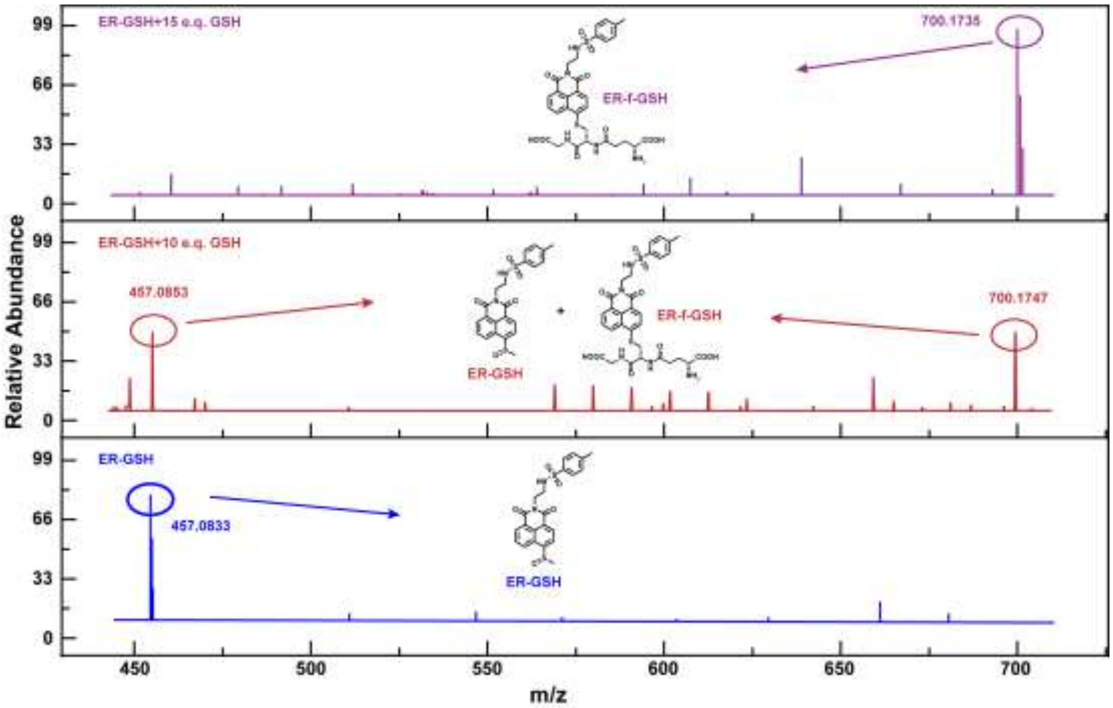

Figure S5. HR-MS spectrum of probe ER-GSH+GSH.

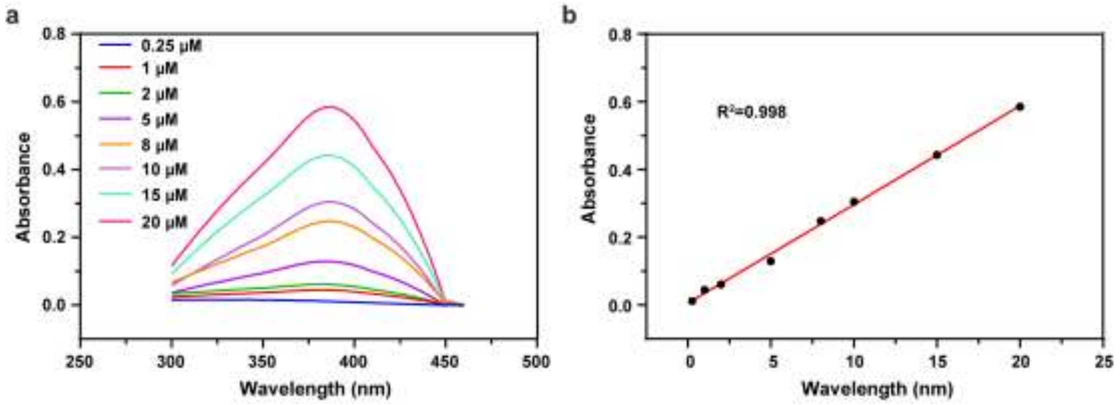

Figure S6. Determination of solubility of ER-GSH.

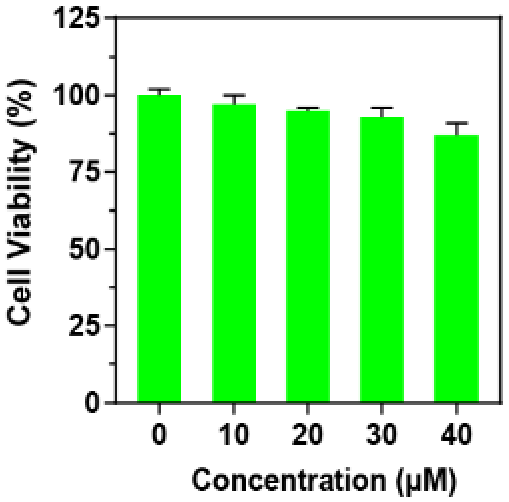

Figure S7. MTT assay of BV2 cells treated with different concentrations of ER-GSH (0, 10, 20, 30, 40 μM).

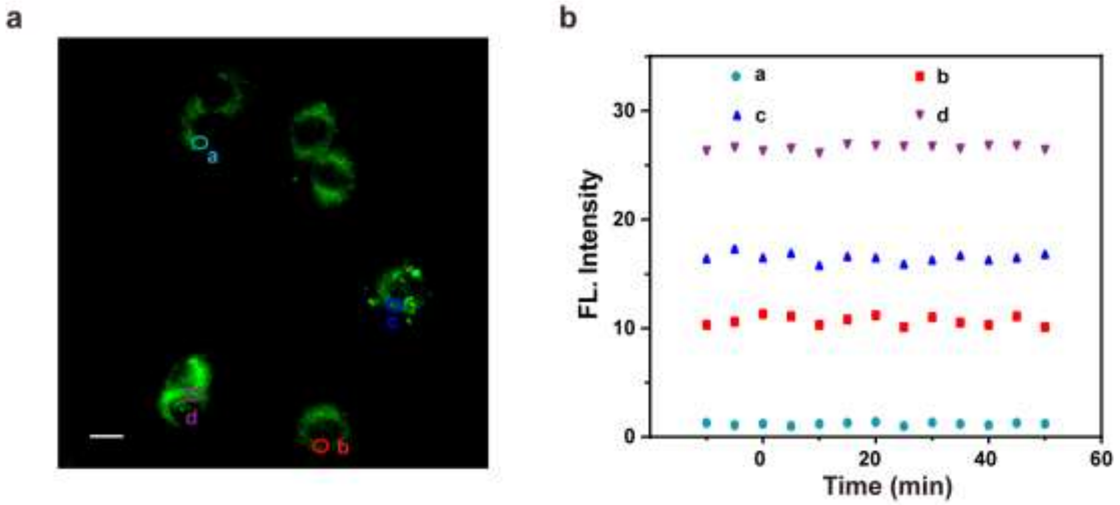

Figure S8. (a) Two-photon fluorescence imaging of ER-GSH (10 μM). (b) The relationship between the fluorescence intensity at points a, b, c and d versus time. Scale bar: 20 μm.

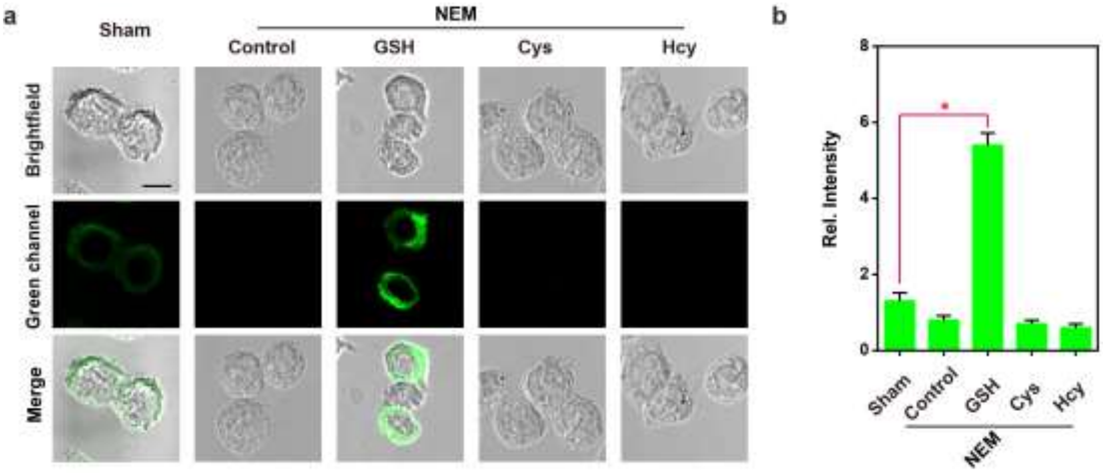

Figure S9. (a) Two-photon fluorescence imaging of ER-GSH alongside endogenous and exogenous GSH in BV2 cells. (b) The histograms indicated relative fluorescence intensity from the corresponding cells. λ_{ex} = 830 nm, λ_{em} = 450–550 nm. Scale bar: 50 μm. The difference was analyzed by one-way ANOVA and Bonferroni post-test. *P < 0.05 *vs* Control group.

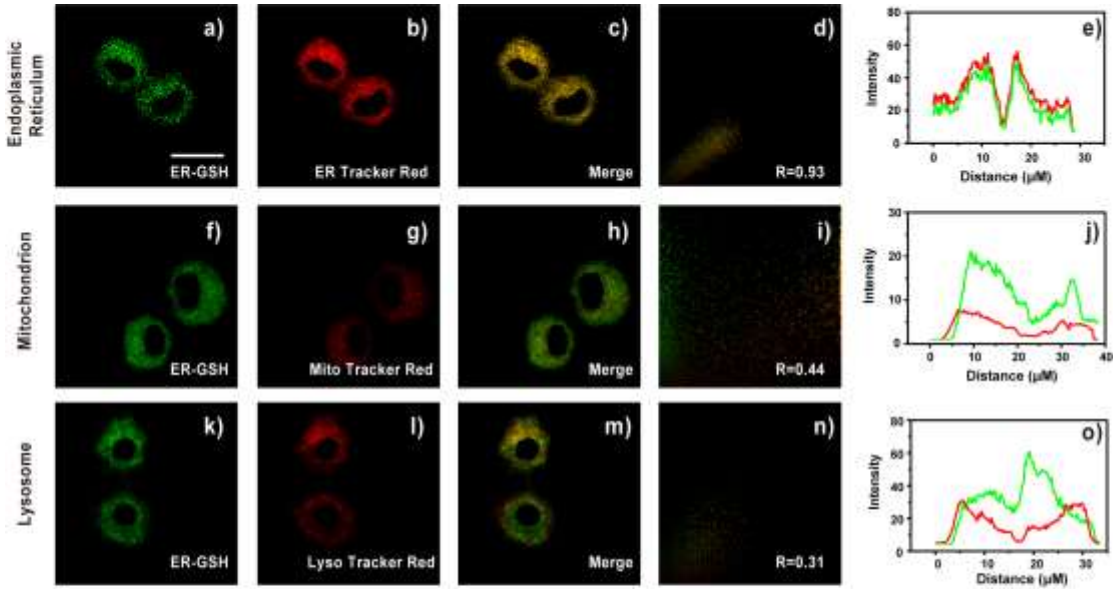

Figure S10. Confocal microscopy images of co-localization BV2 cell imaging of ER-GSH and commercial dyes including (a-c) ER Tracker Red, (e-g) Mito Tracker Red, (i-k) Lyso Tracker Red in BV2 cell. Green channel (450–550 nm, λ_{ex} = 405 nm) for ER-GSH; Red channel (600–640 nm, λ_{ex} = 594 nm) for ER Tracker Red, Mito Tracker Red and Lyso Tracker Red. Scale bar: 50 μm. (d, h, l) Fluorescence intensity correlation plot of ER-GSH and commercial dyes. Scale bar: 50 μm.

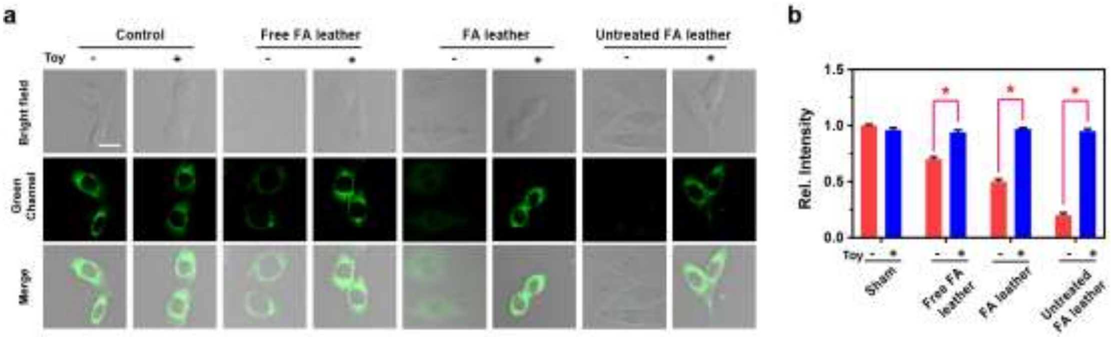

Figure S11. (a) Two-photon fluorescence imaging of ER-GSH in BV2 cells under different conditions. (b) The histograms indicated relative fluorescence intensity from the corresponding cells. The difference was analyzed by one-way ANOVA and Bonferroni post-test. *P < 0.05. Scale bar: 50 μm.

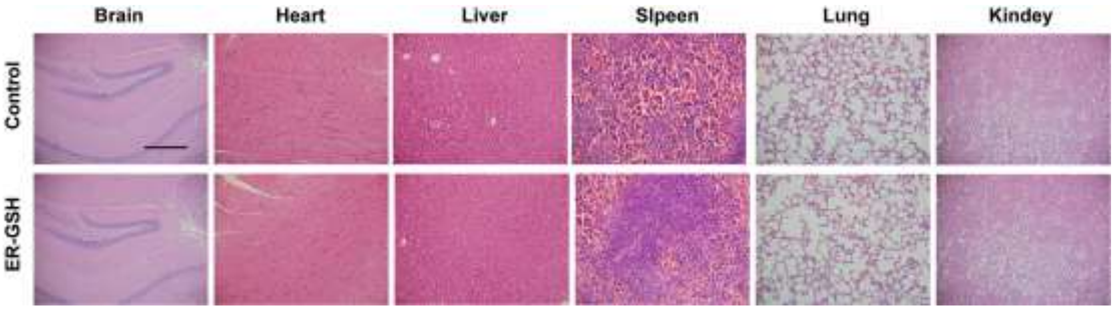

Figure S12. H&E staining results of different organs collected from the control group and ER-GSH (100 μL, 200 μM) treated group. Scale bar: 100 μm.

Table S1 Electronic Excitation Wavelength (nm), Oscillator Strengths (*f*), and Corresponding Compositions for ER-GSH and ER-f-GSH Based on TDDFT Method

	Transition	Wavelength	*f*	Composition	CI	Experimental Wavelength
ER-GSH	$S_0 \rightarrow S_1$	383	0.1642	HOMO→LUMO	0.6610	
	$S_0 \rightarrow S_2$	340	0.1744	HOMO-1→LUMO	0.6490	346
ER-f-GSH	$S_0 \rightarrow S_1$	413	0.3335	HOMO→LUMO	0.6986	388
	$S_0 \rightarrow S_2$	318	0.0005	HOMO-5→LUMO	0.6789	

Table S2 Emission Wavelength (nm) and Corresponding Compositions for ER-GSH and ER-f-GSH Based on TDDFT Method

	Transition	Calculated Wavelength	Experimental Wavelength	Composition	CI
ER-GSH	-	-	-	-	-
ER-f-GSH	$S_1 \rightarrow S_0$	454	488	LUMO→HOMO	0.6991

REFERENCES

(1) Li, S., Zhou, D., Li, Y., Liu, H., Wu, P., Yang, J., Jiang, W., Li, C. Efficient Two-Photon Fluorescent Probe for Imaging of Nitric Oxide during Endoplasmic Reticulum Stress. *ACS Sensors* **2018**, *3(11)*, 2311-2319.

(2) Wang, W., Chai, L., Chen, X., Li, Z., Feng, L., Hu, W., Li, H., Yang, G. Imaging changes in the polarity of lipid droplets during NAFLD-Induced ferroptosis via a red-emitting fluorescent probe with a large Stokes shift. *Biosens. Bioelectron.* **2023**, *231*, 115289.

(3) Wang, Q., Pan, Y., Fu, W., Wu, H., Zhou, M., Zhang, Y. Aminopolycarboxylic acids modified oxygen reduction by zero valent iron: Proton-coupled electron transfer, role of iron ion and reactive oxidant generation. *J. Hazard. Mater.* **2022**, *430*, 128402.

(4) Odyniec, M. L., Park, S.-J., Gardiner, J. E., Webb, E. C., Sedgwick, A. C., Yoon, J., Bull, S. D., Kim, H. M., James, T. D. A fluorescent ESIPT-based benzimidazole platform for the ratiometric two-photon imaging of $ONOO^{-}$ in vitro and ex vivo. *Chem. Sci.* **2020**, *11(28)*, 7329-7334.

(5) Le, A. P., Rupprecht, J. F., Mège, R. M., Toyama, Y., Lim, C. T., Ladoux, B. Adhesion-mediated heterogeneous actin organization governs apoptotic cell extrusion. *Nat. Commun.* **2021**, *12(1)*, 397.

(6) Lai, R., Liu, Y., Luo, X., Chen, L., Han, Y., Lv, M., Liang, G., Chen, J., Zhang, C., Di, D., Scholes, G. D., Castellano, F. N. Wu, K. Shallow distance-dependent triplet energy migration mediated by endothermic charge-transfer. *Nat. Commun.* **2021**, *2(1)*, 1532.

(7) Jin, C., Wu, P., Yang, Y., He, Z., Zhu, H., Li, Z. A novel fluorescent probe for the detection of peroxynitrite and its application in acute liver injury model. *Redox Biol.* **2021**, *46*, 102068.

(8) Zhang, Z. D., Xiong, T. C., Yao, S. Q., Wei, M. C., Chen, M., Lin, D., Zhong, B. RNF115 plays dual roles in innate antiviral responses by catalyzing distinct ubiquitination of MAVS and MITA. *Nat. Commun.* **2020**, *11(1)*, 5536.

(9) Zhao, X., Xie, L., Wang, Z., Wang, J., Xu, H., Han, X., Bai, D., Deng, P. ZBP1 (DAI/DLM-1) promotes osteogenic differentiation while inhibiting adipogenic differentiation in mesenchymal stem cells through a positive feedback loop of Wnt/β-catenin signaling. *Bone Res.* **2020**, *8*, 12.
